# Effect of Left Atrial Pulmonary Vein Angiography on Safety and Efficacy for High-Power, Short-Duration Pulmonary Vein Isolation in Patients with Atrial Fibrillation

**DOI:** 10.3390/jcm12031094

**Published:** 2023-01-31

**Authors:** Sebastian Weyand, Viola Adam, Matthias Beuter, Simon Hanger, David Heinzmann, Willibald Schrezenmeier, Peter Seizer

**Affiliations:** 1 Medizinische Klinik II—Kardiologie und Angiologie, Ostalb-Klinikum Aalen, Im Kälblesrain 1, 73430 Aalen, Germany; 2 Innere Medizin III—Kardiologie und Angiologie, Universitätsklinikum Tübingen, Otfried-Müller-Straße 10, 72076 Tübingen, Germany

**Keywords:** atrial fibrillation, catheter ablation, pulmonary vein isolation, high power short duration ablation, pulmonary vein angiography, preprocedural imaging

## Abstract

Imaging of pulmonary vein (PV) anatomy by angiography before pulmonary vein isolation (PVI) for atrial fibrillation (AF) has long been standard practice in many centers. Nowadays, very accurate anatomical maps can be generated by the use of high-resolution mapping catheters, and very effective ablation lesions can be generated by the use of the high-power, short-duration (HPSD) technique. In our center, PV angiography was routinely performed before PVI. However, since there is no clear evidence for this, we refrained from performing PV angiography. This study aimed to investigate whether PV angiography is still necessary when using high-resolution mapping catheters after ablation in the high-power, short-duration (HPSD) technique. A total of 139 consecutive patients with atrial fibrillation (66.25 ± 11.68 years old, 62.39% male) undergoing radiofrequency PVI were included in the study. Ablation was performed with the HPSD technique using a fixed protocol for energy delivery of 50 watts (contact force 3–20 g). We observed no significant effect on the efficacy, efficiency and complications of the ablation procedure if pulmonary vein angiography was omitted before HPSD PVI. Thus, using our protocol, it may be useful that PV angiography is avoided, especially in young patients and those with chronic renal disease.

## 1. Introduction

With an estimated lifetime risk of 22–26%, atrial fibrillation (AF) is the most common arrhythmia and has substantial population health consequences [1]. Compared with medical therapy alone, catheter ablation of AF has been shown to be an effective procedure and to improve quality of life [2,3]. The main strategy for the ablation of AF is pulmonary vein isolation (PVI) by circular ablation around the pulmonary vein (PV) ostia to isolate trigger foci [4]. For patients with paroxysmal or persistent AF, the current atrial fibrillation guidelines provide a class I recommendation for PVI when drug therapy has failed or when patients have an impaired ejection fraction [5]. High-power, short-duration (HPSD) PVI, as a newer protocol for the radiofrequency ablation of AF, increases procedural efficiency [6] compared to low-power, longer-duration (LPLD) ablation. It has been demonstrated that the use of high-resolution mapping catheters in the ablation of AF can reduce radiofrequency and procedure times compared with a validation set in which a conventional ablation approach was used [7]. For years, imaging of the pulmonary veins by CT, MRI, or PV angiography was performed before PVI because it was thought to facilitate the ablation process and identify the different variants of PV anatomy [8]. Over time, with the development of new mapping technologies, PV angiography has increasingly not been performed; in 2017, only 25% of writing group members of the HRS/EHRA/ECAS/APHRS/SOLAECE expert consensus statement on catheter and surgical ablation of atrial fibrillation routinely used PV venography during their AF ablation procedures [9]. It has been shown that the cryoballoon ablation of atrial fibrillation is effectively feasible without previous imaging of pulmonary vein anatomy [10]. However, to our knowledge, the effect of omitting PV angiography on the efficacy and safety of HPSD PVI has never been studied in a structured manner. To investigate this is the aim of this study.

## 2. Materials and Methods

This retrospective study included 139 consecutive adult patients with symptomatic atrial fibrillation undergoing first-time PVI in HPSD technique at Ostalb-Klinikum Aalen between October 2019 and October 2021. All patients underwent their PVI due to recurrent symptomatic episodes of paroxysmal or persistent AF. Patients were divided retrospectively into either the group that received PV angiography before PVI (Angiography +) or the group that did not receive PV angiography (Angiography −). This study was approved by the ethics committee of the Landesärztekammer Baden-Württemberg. All patients gave informed consent to the ablation procedure and post-ablation diagnostics.

### 2.1. Ablation Procedure

All PVIs were performed according to our previously published Aalen protocol for HPSD-PVI [11]. Transesophageal echocardiography with a special focus on excluding LAA thrombi was performed before the procedure. All patients were anticoagulated at least 3 weeks before and 3 months after the procedure independent of CHA_2_DS_2_-VASc score. Anticoagulation with a DOAK was continued except for a single dose omission on the morning of the procedure, which was instead applied 4 h after the procedure after the exclusion of major bleeding complications. Phenprocoumon was continued, aiming for an International Normalized Ratio (INR) between 2.0 and 3.0. Electrophysiological study ablation was performed in spontaneously breathing patients under deep intravenous sedation with propofol and piritramide. Patients were anticoagulated with intravenous heparin during the procedure. The infusion was adjusted to maintain an activated clotting time of 300–400 s. Detailed electroanatomic data were obtained using either the CARTO (Biosense Webster, Diamond Bar, CA, USA) or Ensite NavX (Abbott Laboratories, Chicago, IL, USA) mapping system. Double transseptal access was obtained. The high-resolution mapping catheter (CARTO Pentaray, Biosense Webster/Advisor HD Grid, Abbott Laboratories) and an open-irrigated and contact force-sensing ablation catheter (THERMOCOOL SMARTTOUCH SF, Biosense Webster/TACTICATH QUARTZ, Abbott Laboratories) were inserted into the left atrium (LA) via transseptal sheaths. In all patients, a high-density electroanatomical map of the LA and PV was obtained to visualize anatomy and determine the extent of LA fibrosis. The high-resolution catheter and, if necessary, the ablation catheter were used to envision all pulmonary veins, with particular emphasis on finding additional pulmonary veins. The procedure was performed as wide antral circumferential point-by-point ablation for isolation of the PV with radiofrequency energy of 50 W and 15 s for each point on the anterior wall and 10 s for each point on the posterior wall. The target contact force ranged from 3 to 20 g. For safety reasons, a temperature probe was placed in the esophagus. After ablation, entrance and exit blocks were demonstrated for each PV and the carinas between PVs with and without administration of 12 mg of intravenous adenosine. If an entry block could not be detected after a simple circular line, detailed mapping was performed with additional ablations at the conduction gap. If AF did not stop spontaneously after ablation, sinus rhythm was restored by external cardioversion. If atrial arrhythmias were sustained after PVI, additional substrates were modified. Cavotricuspid isthmus ablation was performed in patients who had documented typical atrial flutter. Patients stayed in the hospital under continuous rhythm monitoring for at least 24 h.

### 2.2. Pulmonary Vein Angiography

**Figure 1:**
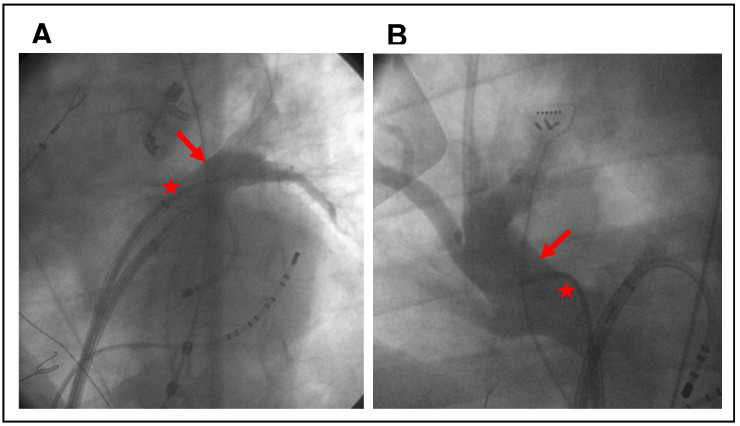
Examples of intraprocedural pulmonary vein angiography (arrows). (**A**) shows the left superior pulmonary vein at 40° LAO, and (**B**) shows the right superior pulmonary vein at 30° RAO. The MP-1 catheter was inserted via a transseptal sheath (asterisks).

In our center, PV angiography was routinely performed before PVI. At that time, there was no subset of patients for whom this was waived. However, since there is no clear evidence for this, we refrained from doing it at a certain point in 2020 after revising our protocol for first-time PVIs. Thus, all patients before this time point received PV angiography, and all after did not. Beyond this historical indicator, there were no other indicators for or against performing PV angiography. If angiography of the pulmonary veins was performed, then a 5-F multipurpose 1 (MP-1) catheter was inserted via the transseptal guiding introducer sheath immediately after the successful transseptal puncture. The individual pulmonary veins were selectively catheterized with the MP-1 catheter, and between 5 and 10 mL of contrast agent was applied to each of the pulmonary veins under fluoroscopy in cine mode (see Figure 1). The anatomy and dimensions of left PVs were assessed in the left anterior oblique (LAO) at 40°, and the anatomy and dimensions of the right PVs were assessed in the right anterior oblique (RAO) at 30° projection. Thereafter, the MP-1 catheter was replaced with the mapping catheter, and a 3D electroanatomic map of the LA was obtained.

### 2.3. Follow-Up

After ablation, each patient was advised to see us immediately if arrhythmias or other symptoms related to AF or heart failure recurred. Due to our standard follow-up protocol for patients receiving a PVI, routine 24-h Holter monitor recordings were performed 6 and 12 months after PVI. The follow-up was carried out over one year. Several major and minor complications were evaluated. Major complications included procedure-related deaths, atrio-esophageal fistulae, procedure-related strokes or TIAs, pericardial tamponades requiring intervention, hemothorax, severe air embolism with ST elevation and hemodynamic collapse, new-onset renal failure requiring dialysis, and vascular access complications requiring surgical intervention. Minor complications included vascular access complications that did not require surgical intervention, acute hypersensitivity reactions to the contrast agent, postinterventional pericarditis and small pericardial effusions that resolved spontaneously without intervention.

### 2.4. Endpoint

We aimed to analyze the impact of pulmonary vein angiography during the HPSD ablation of AF on procedural and safety parameters of catheter ablation and on freedom from AF recurrence after the first PVI. AF recurrences were defined as documented AF or atrial tachycardia (AT) in a Holter ECG with a duration longer than 30 s or in a 12-lead ECG. The endpoint in the time-to-event analysis was the first documented recurrence occurring more than 90 days after the ablation procedure. Recurrences during the 90-day blanking period were not counted for the endpoint analysis as they are frequent and due to the inflammation in the LA or incomplete healing of the ablation lesions. Furthermore, they do not predict long-term outcomes, as up to half of the patients with early recurrence remain AF-free in the long term [12].

### 2.5. Statistical Analysis

Categorical variables are expressed as frequencies and percentages and compared using chi-squared or Fisher’s exact test. All continuous variables were tested for normal distribution. Continuous variables are presented as mean value ± standard deviation and compared using an unpaired two-sided Student’s t-test or Mann–Whitney U test, respectively. Event-free survival was calculated by Kaplan–Meier analysis as time from PVI to the first documented AF/AT episode. The log-rank test was used to assess differences in event-free survival time between groups. Two-sided *p*-values of less than 0.05 were considered statistically significant. Data analysis was performed using Excel 2016 (Microsoft Corporation, Redmond, WA, USA) and GraphPad Prism 9 (GraphPad Software, Inc., San Diego, CA, USA).

## 3. Results

### 3.1. Baseline Characteristics

A total of 139 consecutive patients (66.25 ± 11.68 years old, 62.39% male) with symptomatic atrial fibrillation undergoing HPSD ablation were included in this study. Overall, 67 patients (48.2%) received PV angiography during the ablation procedure, and in 72 patients (51.8%), no PV angiography was performed. Baseline clinical subject characteristics are listed in Table 1. There were proportionally more male patients in the group who received PV angiography. Otherwise, there was no significant difference in age, BMI, AF pattern, CHA_2_DS_2_-VASc score, left ventricular ejection fraction, LA diameter, extent of atrial fibrosis or cardiovascular co-morbidity profile between the two groups. In the majority of patients, normal LA and PV anatomy was found in the electroanatomical map and in the PV angiograms. In 12 patients (8.6%), a PV common ostium was identified, and in 24 patients (17.3%), an additional PV was found. The percentage of PV anomalies was not significantly different between the groups. In contrast, no pulmonary vein abnormalities were described in preprocedural transesophageal echocardiography.

### 3.2. Procedural Data

**Figure 2:**
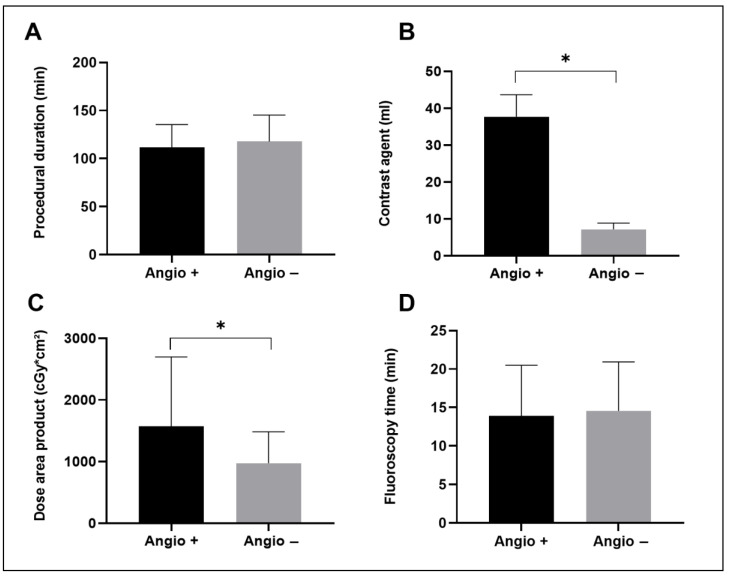
Comparison of selected procedural parameters between patients who had received PV angiography (Angio +) and those without PV angiography (Angio −) (mean with standard deviation). (**A**) shows the procedural duration, (**B**) shows the volume of contrast agent applicated during the whole procedure, (**C**) shows the dose area product for fluoroscopy and (**D**) shows the fluoroscopy time. * indicates statistical significance.

Acute procedural success with complete PVI demonstrated by entrance and exit block for each PV and the carinas between PVs was achieved in all patients. Additional ablations were performed in 13 patients (19.4%) of those who received PV angiography and in 9 (12.5%) of those without PV angiography. In addition to ablations of the cavotricuspid isthmus for typical atrial flutter, this has involved in particular left atrial substrate-based ablations (roof line and anterior mitral line) for sustained atrial fibrillation or atypical atrial flutter after PVI. These left atrial ablations occurred in 8 (11.9%) of the patients in the Angiography + group and 7 (9.7%) in the Angiography − group. The mean procedural skin-to-skin duration was 111.8 ± 23.65 min in the Angiography + group and 117.7 ± 27.60 min in the Angiography − group. The dose area product was significantly larger in the Angiography + than in the Angiography − group (1574 ± 1125 cGy*cm^2^ and 976.1 ± 506.9 cGy*cm^2^, respectively). In addition, significantly more contrast agent was administered in the Angiography + than in the Angiography − group (37.75 ± 5.92 mL and 7.17 ± 1.74 mL, respectively) (Table 2 and Figure 2).

### 3.3. Complications

No major complication requiring intervention occurred in either group. In particular, no procedure-related deaths, atrio-esophageal fistulae, procedure-related strokes or TIAs, pericardial tamponades requiring intervention, severe air embolism with ST elevation and hemodynamic collapse, hemothorax and vascular access complications requiring surgical intervention were observed. Furthermore, there were no cases in either group with acute symptomatic acute renal failure (with and without the need for dialysis) after the intervention. However, laboratory testing of kidney function was not routinely performed in asymptomatic patients after ablation; thus, milder cases with renal function deterioration cannot be excluded with certainty. As minor complications, we observed 1 pseudo-aneurysm and 2 postinterventional pericarditis with pericardial effusion in either group (Table 2). All minor complications were followed up and improved without interventional or surgical intervention.

### 3.4. Clinical Outcome

**Figure 3:**
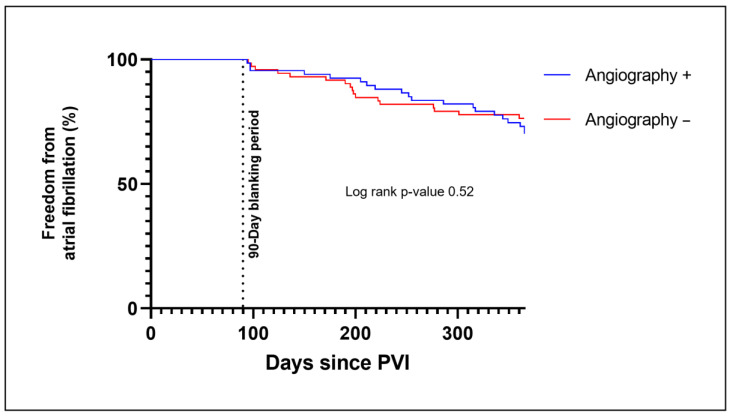
Kaplan–Meier plot for freedom from AF for those patients who received a PV angiography during PVI (Angiography +) and for those without PV angiography (Angiography −). Recurrences of AF during the 90-day blanking period were not counted in the determination of the first clinical failure. Abbreviations: PVI, pulmonary vein isolation.

Within the follow-up period of 1 year, recurrence of AF occurred in 26.6% of patients (20 patients in the Angiography + and 17 patients in the Angiography − group) (Table 2). In the Kaplan–Meier analysis, there was no significant difference in freedom from AF in both groups in the first year after PVI (log-rank *p*-value 0.52) (Figure 3). Among patients who received additional left atrial lines because of sustained atrial fibrillation or atypical atrial flutter after PVI 4 (50%) in the Angiography + and 3 (42.9%) in the Angiography −group showed a recurrence of atrial arrhythmia in the follow up. This is higher than in the overall study population, although the absolute numbers for this subgroup were low in the study.

## 4. Discussion

In this study, we examined the impact of preablational visualization of left atrial PV anatomy by selective angiography on the safety and efficacy of HPSD PVI of AF. Baseline characteristics of patients are representative of patients with atrial fibrillation [9] and in line with those of other trials about HPSD ablation [13]. It was shown that the omission of pulmonary vein angiography before HPSD PVI had no negative impact on the complications as well as the efficiency and success of the ablation procedure but was associated with a significantly lower amount of applied contrast agent as well as reduced radiation dose.

### 4.1. Procedural Data

PV entrance and exit blocks as primary targets of the PVI procedure could be achieved in all patients. The mean skin-to-skin time was 114.8 ± 25.85 min, which is comparable with a recent meta-analysis about the efficiency of HPSD ablation [14]. Although we did not find a statistically significant difference in procedure time and fluoroscopy time between the groups, it is noteworthy that patients who received PV angiography had slightly lower procedure and radiation times than patients without. This may be explained by the fact that although time is saved by omitting angiography, more time is lost for the high-resolution map to visualize all PVs and atypical anatomies with high confidence. In addition, a potential data bias attributable to learning-curve effects cannot be fully excluded as it used to be common practice to perform PV angiography prior to PVI, and only in recent years has preprocedural imaging been more frequently omitted. Thus, it is possible that as mapping experience increases without PV angiography, the time required will become shorter. While the fluoroscopy time in our procedures is comparable to that found by Chun et al. [15] in a collective of 2125 patients who received PVI with radiofrequency energy, no significant difference was found between the patients who received PV angiography and those without PV angiography. However, radiation exposure was significantly reduced by omitting PV angiography, mainly because it was performed in cine mode, in which good image quality is associated with increased radiation, in contrast to the rest of the intervention, which took place in the low-dose mode at 3.75 frames per second. That offers the possibility of reducing harmful DNA damage from radiation exposure [16], which is especially important for young patients receiving PVI. Apart from the contrast agent consumption for the PV angiographies, only a small amount of contrast agent is needed for the transseptal puncture, which explains the significant difference in the amount of contrast agent consumed between the two groups. Even though to our knowledge, there were no cases of clinically relevant acute renal failure in this study, this may be particularly important in patients with chronic kidney disease with an estimated glomerular filtration rate (eGFR) < 45 mL/min/1.73 m^2^, who are at increased risk for contrast-induced nephropathy [17]. In addition, it has been shown that a reduction in the dose of iodinated contrast agents also resulted in a lower rate of acute hypersensitivity reactions [18].

### 4.2. Complications

No periprocedural complications requiring intervention occurred in our study population. Both pseudo-aneurysm and postinterventional pericarditis are known complications after PVI and comparable to other PVI collectives in the extent to which they occurred in this study [19]. An advantage of not performing PV angiography is the reduced risk of air embolism and LA injury with subsequent pericardial tamponade, as there is one less catheter exchange through the transseptal sheath. Catheter manipulation is one main risk factor for cardiac tamponade in PVI [15]. However, this could not be shown in this study, as neither of these complications occurred in either group.

### 4.3. Clinical Outcome

Understanding pulmonary vein anatomy is considered crucial to performing successful PVI and avoiding pulmonary vein stenosis [20]. Marom et al. [21] found, by use of computed tomography (CT) in 201 patients, 28% with more than 4 pulmonary veins and 16% of patients with a single PV ostium. In comparison, in a study of 783 patients who underwent computed tomography pulmonary angiography, pulmonary vein abnormalities were found in only 26 (3.3%) of patients. In our study, we found additional pulmonary veins in 17.3% of patients and a common PV ostium in 8.6% of cases. Since there is no statistically significant difference between groups, detection by mapping alone does not appear to be inferior to additional PV angiography. The use of high-resolution mapping catheters facilitates the generation of very accurate maps of the LA, which can help in the visualization of unusual pulmonary vein anatomies. Moreover, by the use of wide antral circumferential ablation (WACA) for PVI, pulmonary vein stenosis has become extremely rare [22]. Furthermore, in the unlikely case that an additional pulmonary vein is not found by mapping alone, the impact seems to be relatively small, as it should be captured by WACA in most cases since additional veins are mostly right-sided middle lobe veins [21] which are located between the two regular right-sided PV and are thus automatically covered by the ablation line typical with the WACA approach. The recurrence rate of 26.6% within the first year after PVI found in our study was not significantly different between groups and is comparable with data published by Arbelo et al., who found a 12-month success rate of 73.6% after AF ablation. These results indicate that pulmonary vein imaging does not affect the clinical outcome of HPSD PVI. We explain this by the very effective lesions generated with HPSD ablation [6] combined with the safe isolation of various pulmonary vein anatomies by the WACA approach and accurate anatomical map generation and testing of entry and exit blocks by high-resolution mapping catheters.

### 4.4. Other Forms of Preprocedural PV Imaging

In some centers, instead of imaging the PV by angiography, CT or magnetic resonance imaging (MRI) is performed to assess the LA and PV anatomy; however, Strohmer et al. [23] found a good correlation between PV anatomy detected by angiography and CT. Another important point for preprocedural CT or MRI imaging is to identify the relationship between the esophagus and the LA, as injury to the esophagus can result in the fatal complication of an atrio-esophageal fistula. However, as the esophagus can migrate from one side to another during the ablation procedure [24], a real-time assessment of esophageal location, as used in our center by esophageal temperature monitoring, may be more useful in minimizing the risk of esophageal injury than pre-procedural imaging [25]. Other aspects mentioned in the literature that argue for performing preprocedural CT or MRI diagnostics, such as the visualization of LA anatomy and size, the exclusion of thrombi in the left atrial appendage (LAA), and the identification of fossa ovalis abnormalities for planning transseptal puncture [10], can already be clarified by preprocedural transesophageal echocardiography. In this study, none of the PV abnormalities later found on PV angiography or high-resolution mapping were identified by preprocedural transesophageal echocardiography. The focus of echocardiography was specifically to exclude LAA thrombi, so no special views were performed to visualize all pulmonary veins. However, this reinforces the fact that in the group in which no pulmonary vein angiography was performed, the high-resolution map alone was sufficient to ensure adequate visualization of the anatomy to perform safe and effective PVI.

### 4.5. Limitations

There are some limitations to this study. All patients had received transesophageal echocardiography before the procedure, so no conclusion can be drawn as to whether PV angiography can be omitted even without this modality. Since it used to be common practice to perform PV angiography prior to PVI, and only in recent years has preprocedural imaging been more frequently omitted, potential data bias attributable to learning-curve effects cannot be fully excluded. Furthermore, the results are only valid for the combination of HPSD PVI and a high-resolution mapping catheter and cannot be transferred to other radiofrequency PVI settings or cryoballoons. It must be emphasized that this retrospective study in itself may not be adequately powered to demonstrate noninferiority, and thus, it would be desirable to conduct a randomized, prospective study to definitively address this issue.

## 5. Conclusions

This study demonstrated that the omission of preprocedural pulmonary vein imaging by PV angiography was not associated with increased complications or a greater recurrence rate of AF in the first year after PVI using our protocol for HPSD PVI. Because of the lower amount of radiation dose and contrast agent required, it may be useful that PV angiography is avoided, especially in young patients and those with chronic renal disease.

## Figures and Tables

**Table 1:** Baseline Clinical Characteristics (*n* = 139).

	Angiography + (*n* = 67)	Angiography − (*n* = 72)	*p*-Value
Age (years)	66.09 ± 10.54	66.40 ± 12.72	0.48
Male, *n* (%)	48 (71.64%)	39 (53.42%)	0.04 *
BMI (kg/m^2^)	28.31 ± 5.23	28.41 ± 5.17	1.00
Paroxysmal AF, *n* (%)	21 (31.34%)	29 (40.28%)	0.29
CHA_2_DS_2_-VASc score	2.46 ± 1.40	2.42 ± 1.47	0.87
LV ejection fraction %	55.23 ± 12.50	57.36 ± 9.28	0.58
LA diameter, mm	46.88 ± 8.86	46.41 ± 8.40	0.87
Atrial fibrosis %	23.23 ± 26.81	18.92 ± 25.21	0.12
PV common ostium	4 (5.97%)	8 (11.11%)	0.37
Additional PV	9 (13.43%)	15 (20.83%)	0.27
Chronic kidney disease, *n* (%)	12 (17.91%)	14 (19.44%)	0.83
Coronary artery disease, *n* (%)	17 (25.37%)	16 (22.22%)	0.69
Hypertension, *n* (%)	41 (61.19%)	46 (63.89%)	0.86
Diabetes, *n* (%)	8 (11.94%)	13 (18.06%)	0.35
Prior stroke/TIA, *n* (%)	6 (8.96%)	1 (1.39%)	0.06

Data given as *n* (%) or mean ± SD. * and bold letters indicate statistical significance. Abbreviations: BMI, body mass index; AF, atrial fibrillation; LV, left ventricle; LA, left atrium; PV, pulmonary vein; TIA, transient ischemic attack.

**Table 2:** Procedural parameters and clinical outcome of pulmonary vein isolation.

	Angiography + (*n* = 67)	Angiography − (*n* = 72)	*p*-Value
PV entrance and exit block	67 (100%)	72 (100%)	1.00
Additional ablations (substrate and/or CTI)	13 (19.4%)	9 (12.5%)	0.35
Procedural duration (min)	111.8 ± 23.65	117.7 ± 27.60	0.18
Dose area product (cGy*cm^2^)	1574 ± 1125	976.1 ± 506.9	0.01 *
Fluoroscopy time (min)	13.9 ± 6.59	14.56 ± 6.36	0.45
Contrast agent (ml)	37.75 ± 5.92	7.17 ± 1.74	<0.01 *
Major complication	0 (0%)	0 (0%)	1.00
Minor complication	3 (4.41%)	3 (4.47%)	1.00
Recurrence of AF in the first year after PVI	20 (29.85%)	17 (23.61%)	0.45

Data given as *n* (%) or mean ± SD. * and bold letters indicate statistical significance. Abbreviations: PV, pulmonary veins; CTI, cavotricuspid isthmus; AF, atrial fibrillation.

## Data Availability

The data presented in this study are available on request from the corresponding authors.
